# Subclinical vascular, hemodynamic and arterial stiffness changes in adults with cystic fibrosis: cross-sectional observational study

**DOI:** 10.1038/s41598-024-63904-0

**Published:** 2024-06-07

**Authors:** Bruno Porto Pessoa, Marcelo Velloso, Érika Pereira Inácio, Claudineia de Oliveira Otoni, Marcelo Bicallho de Fuccio, Bruno Almeida Rezende, Maria Glória Rodrigues-Machado

**Affiliations:** 1 Post-Graduate Program in Health Sciences of Medical Sciences Faculty of Minas Gerais, Alameda Ezequiel Dias, 275-Centro, Belo Horizonte, MG 30130-110 Brazil; 2 Adult Cystic Fibrosis Center, Júlia Kubitschek Hospital–FHEMIG, Rua Dr. Cristiano Rezende, 2745–Milionários, Belo Horizonte, MG 30610-720 Brazil; 3 Rehabilitation Sciences Program, Federal University of Minas Gerais, Avenida Antônio Carlos 6627. Pampulha, Belo Horizonte, MG 31270-901 Brazil; 4 Department of Physical Therapy, Federal University of Minas Gerais, Avenida Antônio Carlos 6627. Pampulha, Belo Horizonte, MG 31270-901 Brazil

**Keywords:** Cystic fibrosis, Hemodynamics, Vascular stiffness, Cardiovascular diseases, Physiology, Circulation, Respiration

## Abstract

Cardiovascular diseases can be an emerging complication in cystic fibrosis (CF), as the median life expectancy has improved considerably. The objective of this study was to compare vascular, hemodynamic parameters and arterial stiffness in adult CF patients with healthy participants pared by sex and age, and to assess the factors associated with arterial stiffness in the CF group. This is a cross-sectional observational study. The evaluation of cardiovascular parameters was performed non-invasively using Mobil-O-Graph. 36 individuals with CF and 35 controls were evaluated. The mean arterial pressure (96.71 ± 10.98 versus 88.61 ± 7.40 mmHg, *p* = 0.0005), cardiac output (4.86 ± 0.57 versus 4.48 ± 0.44 L/min, *p* = 0.002) and systolic volume (64.30 ± 11.91 versus 49.02 ± 9.31 ml, *p* < 0.0001) were significantly lower in the CF group. The heart rate was higher in the CF when compared to the control (77.18 ± 10.47 versus 93.56 ± 14.57 bpm, *p* < 0.0001). The augmentation index (AIx@75) was higher in the CF than control (29.94 ± 9.37 versus 16.52 ± 7.179%, *p* < 0.0001). In the multivariate model controlled by body mass index and Forced Expiratory Volume in the first second, central systolic blood pressure and reflection coefficient directly related to AIx@75. Negatively related to AIx@75 were age and systolic volume. The adjusted determination coefficient was 87.40%. Individuals with CF presented lower arterial blood pressures and changes in cardiac function with lower stroke volume and cardiac output. The AIx@75, an indirect index of arterial stiffness and direct index of left ventricular overload, is increased in this population. The subclinical findings suggest the need for earlier cardiovascular assessment in this population due to increased risks of cardiovascular disease.

## Introduction

In the last decades, with the advent of new therapeutic modalities, life expectancy has improved considerably for the population with cystic fibrosis (CF). The number of adults with this disease has grown worldwide^1^. This demographic change from a classically child disease to a predominantly adult one brought new challenges to specialized CF centers, considering that new comorbidities with a possible negative impact on survival will become prevalent and need to be monitored^2,3^.

Cardiovascular diseases (CVD) can be an emerging complication in CF^3^. In addition to older age, classic systemic changes caused by the disease, such as: inflammation, oxidative stress, endothelial dysfunction, diabetes, and chronic hypoxemia, suggest a predisposition for the development of CVD^4^. Although most studies focus on right ventricular dysfunction^5^, several changes in the left side of the heart have already been described in CF^6^.

Arterial stiffness is a parameter that has been widely studied as a predictor of cardiovascular outcomes in several diseases. It is characterized by changes in the physical properties of the vessels, being considered a natural characteristic of aging, but which can have an earlier onset in cases of diseases^7^. Pulse Wave Velocity (PWV) is the most used parameter in clinical practice to assess arterial stiffness. It represents the time taken by the pressure wave to travel through a given segment of the arterial tree^8^. In addition, several important information about the magnitude of the reflected wave and central blood pressure values can be obtained through the analysis of the aortic pressure wave^9^. The Augmentation Index (AIx) is an indirect marker of arterial stiffness and represents the percentage of central pulse pressure (cPP) increase due to the intensity of the reflected wave^10^. Both PWV and AIx are considered independent predictors for CVD^11^. Despite being scarce, previous studies have revealed these CF changes in both children and adults^12–14^. However, no studies simultaneously evaluating stiffness indexes with vascular and hemodynamic parameters were found.

Thus, it is plausible to affirm that the number of CF patients with CVD will increase in the coming years. The identification and early modification of risk factors is the best way to ensure quality of life and survival. Therefore, the objective of this study was to evaluate vascular, hemodynamic parameters and arterial stiffness indices  in adult CF patients using the brachial artery oscillometric method to compare them with healthy participants, and additionally to assess the factors associated with arterial stifness in the CF group.

## Materials and methods

This was a cross-sectional observational study involving patients aged 18 years or older with a CF; diagnosis confirmed by genetic testing with two pathogenic mutations for the disease, no known history of heart disease, clinically stable (absence of new symptoms or decreased Forced Expiratory Volume (FEV_1_) greater than 10% compared with previous spirometry), and in outpatient monitoring at the CF reference center of a public hospital in Belo Horizonte, Brazil. Excluded from the study were: diabetic patients, patients using systemic corticosteroids, and smokers. The CF group participants were compared with healthy participants, without known risk factors for CVD, and no history of lung disease, paired by age and sex. The study was approved by the ethics committee of the Federal University of Minas Gerais and written (CAEE: 88462518.0.0000.5149, appoval number 3.282.533), informed consent was obtained from each participant.

### Study design

The participants were evaluated in a single step. Initially, demographic data including: sex, age, weight, height and Body Mass Index (BMI), were collected. The CF group underwent spirometry and the following data were collected from the medical record: genetic mutations, usual medications, airway colonization, and sweat test value. After ten minutes of rest, vascular and hemodynamic parameters, and arterial stiffness indices were measured in both groups.

### Evaluation of the pulmonary function

CF participants underwent lung function assessment using a previously calibrated Koko spirometer (PDS Instrumentation Inc., Louisville, CO, USA). All tests were performed according to the ATS/ERS standard^15^. Reference values for the Brazilian population were used to calculate the predicted values^16^. The variables evaluated were: Forced Vital Capacity (FVC), forced expiratory volume in 1 s (FEV_1_), FEV_1_/FVC ratio and Peak Expiratory Flow (PEF).

### Evaluation of vascular and hemodynamic parameters and arterial stiffness

The non-invasive evaluation was performed using the Mobil-O-Graph Pulse Wave Analysis Monitor device (Mobil-O-Graph, IEM, Stolberg, Germany) incorporated with the ARCSolver method (Austrian Institute of Technology, Vienna, Austria), which can reconstruct the central or aortic pulse wave from brachial oscillometric pressure using a transfer function^17,18^.

The arterial stiffness indeces evaluated were PWV and AIx@75 (AIx normalized to a heart rate of 75 bpm). PWV was determined using a mathematical model, considering several parameters in the wave of pulse and wave separation analysis. The AIx@75 was evaluated from the wave of aortic pressure through augmentation pressure (pressure difference between the peak of the reflection wave (P2) and the peak of the incident wave (P1)), expressed as the percentage of cPP [AIx@75 = (P2 − P1)/cPP × 100]. In addition to arterial stiffness indeces, the peripheral Systolic Blood Pressure (pSBP), Diastolic Blood Pressure (pDBP), Pulse Pressure (pPP) and Mean (MAP) and central (cSBP, cDBP, cPP) arterial blood pressures were evaluated. The hemodynamic parameters assessed were: Systolic Volume (SV), Cardiac Output (CO), Total Vascular Resistance (TVR), Cardiac Index (CI), and Heart Rate (HR). All measurements were conducted in triplicate and the mean of the three acceptable measurements was considered for the final analysis of all evaluated parameters^19^.

### Statistical analysis

Qualitative variables were presented as absolute and relative frequencies, and quantitative variables as mean ± standard deviation. The quantitative variables were subjected to the Shapiro–Wilk normality test. The groups were compared using the Student’s t or the Wilcoxon tests for independent samples. The Pearson or Spearman correlation tests were used to evaluate bivariate associations. A binary logistic model was built considering the groups (CF and control) as response and AIx@75 and systolic volume as covariates, in order to assess the association between the AIx@75 and the groups controlled by the systolic volume. The results were showed as odds ratio (OR) and 95% confidence intervals (CI).

A multivariate model regarding AIx@75 in the CF group was built. All the variables were included in a full model linear regression model, and by applying backward strategy the final model was assessed, keeping BMI and FEV_1_ even without significance for control. The results were shown as coefficients and 95% confidence intervals. The quality of the adjustment was evaluated by the determination coefficient (R^2^) and residual analysis. The analysis was developed in the software R version 4.3.2, and *p* < 0.05 was considered significant.

### Sample size calculation

The sample size was calculated in order to compare means of AIx@75 between CF and control groups. Under 5% of significance, minimum power of 80%, considering a large effect size (0.7), it was necessary at least 34 participants in each group, in a total of 68 participants in the sample size. Sample size calculation was made in the G*Power 3.1.9.7 software.

### Ethical approval and consent to participate

This study was performed in line with the principles of the Declaration of Helsinki. Approval was granted by the Research Ethics Committee of Federal University of Minas Gerais (protocol n. 88,462,518.0.0000.5149, approval report n. 3.282.533).

## Results

This study included 36 CF patients (24 men) and 35 healthy controls (23 men). The groups were similar regarding age and sex. However, the CF group showed weight, height, and BMI significantly lower than the control group (Table 1).

**Table 1:** Anthropometric data of the participants included in the sample.

Characteristics	Control (n = 35)	CF (n = 36)	*P*-value
Sex (M:F)	23:12	24:12	
Age (years)	31.09 ± 11.69	31.31 ± 11.05	0.83
Height (m)	1.70 ± 0.08	1.65 ± 0.08	0.02
Weight (kg)	65.45 ± 10.11	57.58 ± 13.67	0.0003
BMI (kg/m^2^)	22.57 ± 2.53	20.93 ± 3.63	0.0039

Data presented as mean ± SD. M: male. F: Female. BMI: Body mass index.

Patients in the CF group presented moderate obstructive ventilatory disorder. Three patients (8.33%) had FEV_1_ 80% of the predicted level, eight (22.22%) between 80 and 60%, 13 (36.11%) between 60 and 40%, and 12 (33.33%) had FEV_1_ ≤ 40%. Genetic analysis showed that 19 (52.77%) patients had at least one allele for the F508del mutation and 35 (97.22%) had chronic airway colonization. As for medications, all patients regularly used dornase alpha, 22 (61%) used inhaled antibiotic therapy, and 25 (69.44%) had pancreatic insufficiency and used enzymes. Of the 36 charts analyzed, 31 (86%) had information on the chloride sweat test, with a mean of 100.53 ± 18.82 mmol/L (Table 2).

**Table 2:** Characterization of the CF group.

Characteristic	CF (n = 36)
Spirometry	
FVC (L)	2.76 ± 0.97
FVC% of predicted	66.33 ± 21.15
FEV_1_ (L)	1.77 ± 0.80
FEV_1_% of predicted	49.36 ± 20.85
FEV_1_/FVC	0.63 ± 0.11
PEF (L/min)	346.17 ± 110.94
Mutation	
Homozygous F508del	3 (8.34%)
Heterozygous F508del	16 (44.44%)
Others	17 (47.22%)
Usual medications	
Dornase alfa	36 (100%)
Inhaled antibiotic	22 (61.11%)
Pancreatic enzyme	25 (69.44%)
Airway colonization	
*Pseudomonas aeruginosa*	15 (41.66%)
*Staphylococcus aureus*	10 (27.78%)
*Pseudomonas aeruginosa* and* Staphylococcus aureus*	10 (27.78)
Non-colonized	1 (2.78%)
**Sweat test** (mEq/L)	100.53 ± 18.82

The data is presented in mean ± SD for the spirometry variables and in absolute and relative frequency for the sweat test and other parameters. FVC: Forced vital capacity; FEV_1_: Forced expiratory volume in the first second; PEF: Peak expiratory flow.

**Figure 1:**
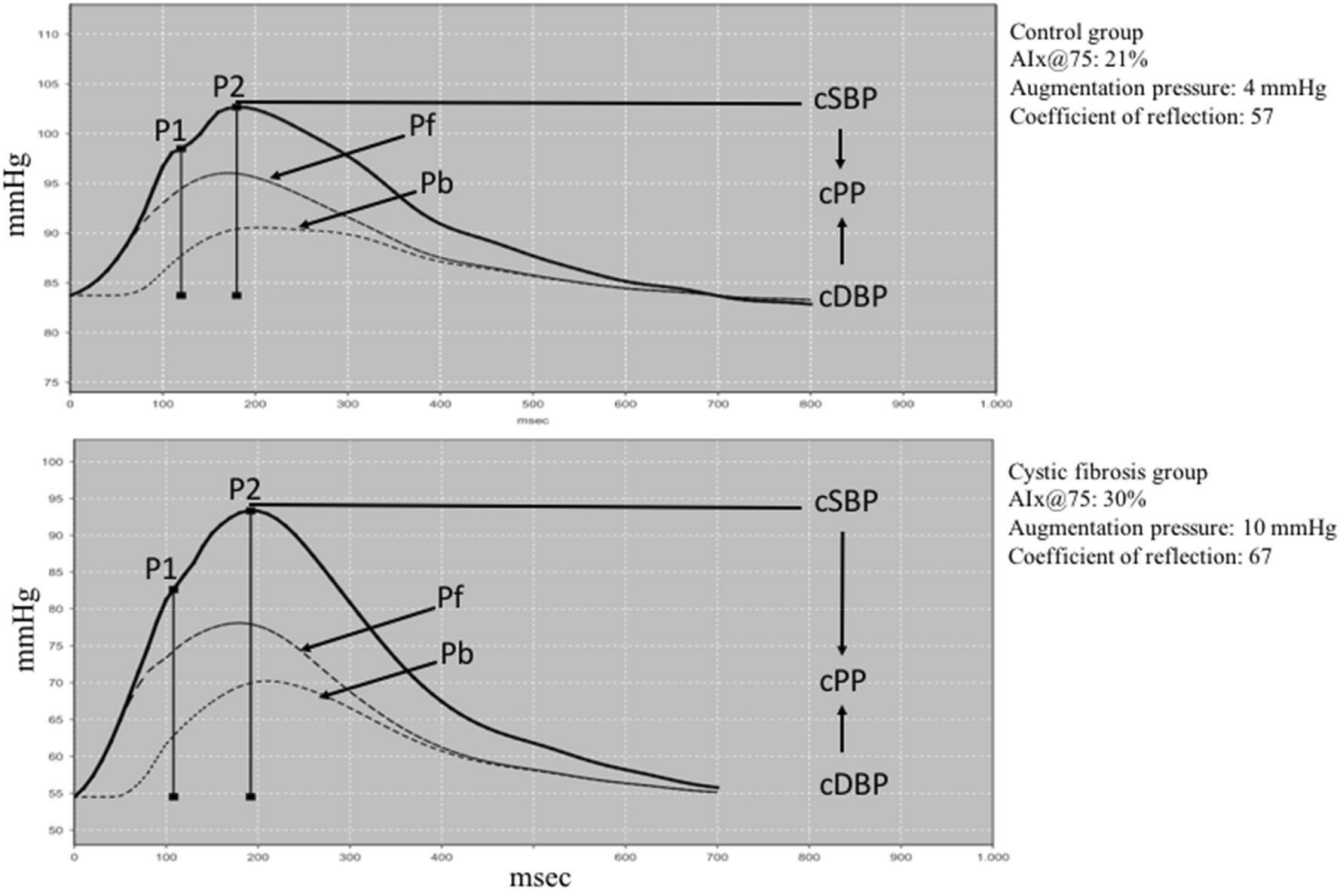
Aortic pulse wave of a control group participant (top) and a cystic fibrosis group participant (bottom). P1 = first systolic peak. P2 = second systolic peak. Pf = forward or ejection wave. Pb = backward or reflexion wave. cSBP and cDBP = central systolicc and diastolic blood pressure. cPP = central pulse pressure. The AIx@75, the augmentation pressure (P2−P1), and the coefficient of reflection (Pb/Pf) are higher in the patient with cystic fibrosis. AIx@75 = (P2 − P1) / cPP *100 normalized for a heart rate of 75 bpm.

Table 3 shows the comparison between vascular and hemodynamic parameters and arterial stiffness indeces between groups. SBP and DBP values, both central and peripheral, MAP, cardiac output and systolic volume were significantly lower in the CF group compared to the control group. On the other hand, HR, AIx@75, the coefficient of reflection, and the augmentation pressure were significantly higher in the CF group. The association between AIx@75 and the groups controlled by the systolic volume was evaluated, and the effect remained significant (OR 1.18, CI 95% 1.04; 1.35) with* p* = 0.015. The other parameters were similar between the groups. Figure 1 shows the aortic pressure wave of a control group participant (top) and a CF group participant (bottom), measured using the Mobil-O-Graph.

**Table 3:** Comparison of peripheral and central blood pressure values, hemodynamic variables, and arterial stiffness in CF and control group participants.

Variables	Control (n = 35)	CF (n = 36)	*P*-value
Peripheral blood pressure (mmHg)			
Systolic blood pressure	119.10 ± 13.14	110.10 ± 7.58	0.0008
Diastolic blood pressure	77.58 ± 11.25	70.54 ± 9.09	0.0048
Mean blood pressure	96.71 ± 10.98	88.61 ± 7.40	0.0005
Pulse pressure	41.87 ± 10.86	39.60 ± 7.80	0.55
Central blood pressure (mmHg)			
Systolic blood pressure	108.9 ± 13.64	100.10 ± 8.51	0.0018
Diastolic blood pressure	79.39 ± 11.99	71.93 ± 8.78	0.0038
Pulse pressure	29.51 ± 7.91	28.19 ± 5.46	0.41
Pulse pressure amplification	1.41 ± 0.14	1.41 ± 0.13	0.86
Hemodynamics parameters			
Systolic volume (ml)	64.30 ± 11.91	49.02 ± 9.31	< 0.0001
Cardiac output (L/min)	4.86 ± 0.57	4.48 ± 0.44	0.0025
Total vascular resistance (s*mmHg/ml)	1.19 ± 0.13	1.19 ± 0.10	0.95
Cardiac index (L/min/m^2^)	2.77 ± 0.29	2.77 ± 0.28	0.89
Heart rate (bpm)	77.18 ± 10.47	93.56 ± 14.57	< 0.0001
Arterial stiffness			
Augmentation pressure (mmHg)	4.88 ± 2.38	5.90 ± 2.18	0.037
Coefficient of reflection	59.08 ± 6.81	62.89 ± 6.03	0.014
AIx@75 (%)	16.52 ± 7.19	29.94 ± 9.37	< 0.0001
PWV (m/s)	5.65 ± 1.60	5.21 ± 0.94	0.24

Data presented in mean ± SD. AIx@75: Augmentation index normalized for a heart rate of 75 bpm; PWV: Pulse wave velocity.

The multiple regression model for AIx@75, as dependent variable, is shown in Table 4. The bivariate associations showed that the AIx@75 was negatively associated with weight (r =  − 0.33 and *p* = 0.045), BMI (r =  − 0.38 and *p* = 0.022), FEV_1_% (r =  − 0.35 and *p* = 0.037), and PEF (r =  − 0.41 and *p* = 0.012).

**Table 4:** Multivariate model regarding AIx@75 for CF patients.

Characteristics	Coefficient	95% CI	*P*-value
Constant	35.03	(18.00; 52.05)	< 0.001
Age	− 0.15	(− 0.28; − 0.02)	0.029
BMI	0.02	(− 0.42; 0.46)	0.925
cSBP	0.22	(0.03; 0.41)	0.023
Systolic volume	− 0.95	(− 1.11; − 0.80)	< 0.001
Coefficient of reflection	0.40	(0.17; 0.62)	0.001
FEV_1_	− 0.63	(− 2.65; 1.24)	0.497

Adjusted determination coefficient was 87.40%. The residuals were normally distributed, homoscedastic and without outliers. BMI: Body mass index; cSBP: Central systolic blood pressure; FEV_1_: Forced expiratory volume in the first second

In the multivariate model controlled by BMI and FEV_1_, cSBP (0.22, 95% CI 0.03; 0.41) and coefficient of reflection (0.40, 95% CI 0.17; 0.62) directly related to AIx@75. Negatively related to AIx@75 were age (− 0.15, 95% CI − 0.28; − 0.02) and systolic volume (− 0.95; 95% CI − 1.11; − 0.80). The adjusted determination coefficient was 87.40% and the residuals were normally distributed, homoscedastic, and without outliers.

## Discussion

To our knowledge, this was the first study that simultaneously evaluated vascular and hemodynamic parameters and arterial stiffness indeces in adult CF patients. The main results of this study show that stable CF adults present higher arterial stiffness evaluated by AIx@75 when compared with controls paired by sex and age. Weight, BMI, FEV_1_% and PEF were negatively associated with AIx@75. In addition, AIx@75 discriminated CF patients with high sensitivity and specificity. These results suggest that CF patients have subclinical changes that can lead to the development of CVD. There were also lower rates of central and peripheral pressures, cardiac output, and systolic volume, and higher heart rate in the CF group compared to the control group.

AIx@75 is an indirect marker of arterial stiffness and represents the magnitude of the reflected wave^10^. Changes in the physical properties of the arteries (vascular aging) may be accelerated in chronic inflammatory diseases such as CF. In the present study, AIx@75 was higher in the CF group compared to controls. Similar results were previously published. Hull et al. (2009) reported higher AIx@75 levels in 50 adult CF patients compared to the control. The increased AIx@75 was independent of confounding factors such as age, sex, height, and pMAP^14^. The arterial stiffness process seems to start in childhood. A recent study showed that arterial stiffness is also present in children even in the absence of risk factors such as hypercholesterolemia, hypertension, and obesity^13^. In the present study, AIx@75 was negatively associated with weight, BMI, FEV_1_%, and PEF. In the final multivariate model controlled by BMI and FEV_1_, cSBP and reflection coefficient directly related to AIx@75. On the other hand, age and systolic volume negatively related to AIx@75. Together, these variables explained 87.40% of the increase in AIx@75 in the CF group.

The systemic inflammatory process of the disease can play an important role in increasing AIx@75 in CF^14,20^. A study with 27 CF children in infectious exacerbation showed a significantly decreased AIx@75 (44.25 ± 13.02% versus 33.48 ± 12.65%) after approximately 14 days of antibiotic therapy. In addition, this decreased AIx@75 remained for one month after treatment. According to the authors, the decreased AIx@75 was due to a decreased inflammatory process, which was assessed by C-reactive protein levels^21^. The present study did not directly measure inflammatory markers, but some clinical characteristics of the sample that are also markers of disease severity and related to the degree of systemic inflammation such as weight, BMI, FEV_1_ and PEF were negatively associated with AIx@75. These results suggest that preventing the recurrence of exacerbations may reduce the risk of cardiovascular complications in this population.

CFTR protein deficiency, which is expressed in endothelial cells and vascular smooth muscle, seems to play a direct role in arterial stiffness, increasing vascular tone^4^ and, consequently, AIx. Adam et al. evaluated the effect of CFTR protein restoration on vascular smooth muscle function in CF adults, reporting a significantly decreased AIx@75 and PWV after 48 h of using ivacaftor, a drug that acts to enhance CFTR protein functioning^22^. These authors also observed improved lung function, lower airway distensibility, and decreased hyperinflation. These direct effects on the airways, together with the systemic effects on the vasculature, suggest vasodilator effect mediated by CFTR protein restoration. The patients in the present study have little CFTR activity, demonstrated by the high sweat test value (100.53 ± 18.82 mmol/L) and the large number of patients with pancreatic insufficiency (69.44%), which may have increased AIx@75 in this population.

AIx@75 is a complex variable and its increase may be due to several mechanisms. It is known that this index is indirectly related to HR, and directly related to PWV and the magnitude of the reflected wave^10^. As recommended, in the present study the AIx values were adjusted to a heart rate of 75 bpm to prevent this variable from interfering with the results. PWV was similar in both groups, but the magnitude of the reflected wave was significantly greater in the CF group. The magnitude of the reflected wave is calculated by the ratio between the amplitude of the reflection wave and the ejection wave, known as the coefficient of reflection. Such findings suggest that the reflected wave was influenced more by the properties of the small arteries than by the elasticity of the aorta since the coefficient of reflection represents the sum of the waves reflected in the bifurcations and in the resistance arteries^23^.

AIx@75 is the most sensitive marker of arterial aging in younger populations, as is the case of the sample in this study. A study with 4001 healthy people in different age groups showed that increases of 9 and 10% in AIx for young men and women, respectively, represent vascular aging equivalent to a decade^11^. In addition, a systematic review that gathered data from 5648 people showed that a 10% increase in AIx increases the risk of cardiovascular events by 31.8% and mortality by 38.4%^24^. The results of the present study showed that the mean AIx@75 in the control group was 13.42% (in percentage terms this increase correspond to 81.23%) higher than the control group. These results suggest that the vascular age of our CF patients is well beyond the chronological age, and that they have an increased risk of cardiovascular events compared to healthy people in the same age group.

Although AIx@75 was significantly higher in the CF group compared to the control group, PWV was similar between the two groups. This difference in responses can be attributed to the determining factors of each of these arterial stiffness indices. AIx@75 depends on the propagation speed of the ejection wave, the amplitude of the reflected wave, the reflection point, and the duration and pattern of ventricular ejection, especially concerning changes in heart rate and ventricular contractility^25^. In contrast, PWV is determined by the propagation speed of the ejection wave from the contraction of the left ventricle and represents intrinsic arterial stiffness. Pathophysiological conditions and medications can alter AIx@75 without altering aortic PWV, suggesting a predominant effect on reflection wave, heart rate, or ventricular ejection, and no change in aortic stiffness^26,27^. Furthermore, AIx@75 and aortic PWV are differentially affected by aging. Changes in AIx@75 are more prominent in younger individuals (< 50 years), whereas changes in aortic stiffness per se are more marked in older individuals (> 50 years), suggesting that AIx@75 might be a more sensitive marker of arterial aging in younger individuals, and aortic PWV more sensitive in those over 50 years of age^28^. As AIx@75 rises steeply with age until 50 years, and aortic PWV after 50 years, the increase in the augmentation pressure is due to an increase in the magnitude of wave reflection rather than increased wave velocity^28^. AIx@75 is mainly associated with peripheral arterial resistance^29^, which is determined primarily by the elasticity of small arteries and arterioles. Our results are in line with this statement, as the augmentation pressure and the coefficient of reflection were significantly higher in the CF group.

The results of the present study show that arterial hypertension does not precede arterial stiffness, since central and peripheral blood pressure levels were significantly lower in the CF group compared to the control group. These findings corroborate the literature^30,31^. Blood pressure is determined by cardiac output and peripheral vascular resistance. As the TVR was similar in both groups, the lower CO in the CF group can explain this finding. The mutation found in CF patients plays a protective role in the cardiovascular system regarding arterial hypertension, which can be explained by the high sodium excretion that occurs through sweat^30,31^. There may also be an attenuation of arterial tone in these patients in the face of sympathetic stimulation. A recent study on animals showed that the F508del expression interferes with the mobilization of calcium in smooth muscle cells, decreasing blood pressure and aortic contractility without decreasing the volume of circulating blood. The authors demonstrated a significant decrease in the responsiveness of isolated aorta in mice stimulated with high doses of norepinephrine, explaining the decreased blood pressure in rats with delta F508del mutation compared to control^32^. A cohort of CF women presented lower SBP and DBP levels and a lower tendency for increased blood pressure with age in relation to the control group. In addition, an inverse relationship was observed between blood pressure and the CFTR functioning^31^, which may justify the findings of the present study, given the low functioning CRTR in the assessed population, as mentioned earlier.

In the present study, heart rate was higher and systolic volume and cardiac output were lower in the CF group. Although the cardiac output is lower in the CF group, it is plausible that there is no impairment in peripheral perfusion, since the cardiac index was similar in both groups. There seems to be an increased heart rate to compensate for the decreased systolic volume. According to the literature, when compared to the control, CF patients have extra 15 beats per minute^33^. A very similar result was observed in the present study. The CF group showed a mean of 17 bpm more than the control group. It is common to find, in the literature, higher resting heart rate values in adults with cystic fibrosis considered clinically stable compared to their healthy peers^34–38^. Higher heart rate levels in CF are multifactorial and may be caused by anemia, hypoxemia, hypercapnia, cachexia, hyperthyroidism, sinus tachycardia and dysfunction of the autonomic nervous system^39,40^. According to the annual exams of our sample, the mean hemoglobin value was 14.19 ± 1.5 g/dl and the mean peripheral oxygen saturation (SpO_2_) was 94.56 ± 2.57% in the cystic fibrosis group, ruling out anemia and hypoxemia as causes of patients’ high heart rate. Cachexia can also be a cause of increased heart rate. However, none of the patients in the present study had a BMI below 18.5 kg/m^2^. In our sample, there was no record of sinus tachycardia or hyperthyroidism in the patients’ medical records. We believe that the increased heart rate in the CF group is due to the decrease in the functioning CFTR protein and its consequences in the body. The literature shows that the use of drugs that correct this protein reduces the resting heart rate of individuals with CF^41^. Furthermore, another study demonstrated that the CF genotype and not circulating catecholamines influence the cardiovascular function of patients^36^.

The cause of lower systolic volume in CF patients is not yet well established. There seems to be a subclinical cardiac dysfunction in patients with CF considered more genetically severe^42^. In addition, the lower systolic volume may be due to changes in contractile characteristics of the heart. A recent study showed that a reduced function of the right and left ventricles may be present in the childhood of clinically stable patients with CF, suggesting that these subclinical cardiovascular changes can generate cardiovascular disease in adulthood^12^.

### Study strengths and limitations

A strong point of this study is the sample. Although it is a sample by convenience in a single center, the hospital is a reference in CF and receives patients from all over the state, and the power of the main result of the study was 96.27%. This study has several limitations. One of them was the impossibility of verifying the association of AIx@75 with inflammatory markers, known as predictors of increased arterial stiffness in several health conditions. Some studies showed that treatment with anti-inflammatory stabilizes lung inflammation, prevents extrapulmonary complications, and reduces accelerated vascular aging^14^. In addition, the patients were not categorized into genetic classes due to the great mutation variability, making it impossible to check whether the CFTR protein is involved in hemodynamic and arterial stiffness changes in that population. Lastly, according to A Scientific Statement from The American Heart Association^7^ , single-point estimates of pulse wave velocity are not recommended because there is a lack of evidence of cardiovascular outcome prediction in longitudinal studies.

## Conclusion

CF patients showed lower levels of central and peripheral vascular pressures, as well as systolic volume and cardiac output. The AIx@75, which is an indirect index of arterial stiffness and direct index of left ventricular overload, is increased in this population even in the presence of lower cSBP. The subclinical findings of this study suggest the need for earlier cardiovascular assessment in this population due to increased risks of cardiovascular disease.

## Data Availability

The datasets used and/or analysed during the current study are available from the corresponding author on reasonable request.

## Abbreviations

AIx: Augmentation Index
AIx@75: Augmentation index normalized to a heart rate of 75 bpm
BMI: Body mass index
cDBP: Central diastolic blood pressure
CF: Cystic fibrosis
CI: Cardiac index
CO: Cardiac output
cPP: Central pulse pressure
cSBP: Central systolic blood pressure
CVD: Cardiovascular diseases
FEV_1_: Forced expiratory volume in the first second
FVC: Forced vital capacity
HR: Heart rate
MAP: Mean arterial pressure
pDBP: Peripheral diastolic blood pressure
PEF: Peak expiratory flow
pPP: Peripheral pulse pressure
pSBP: Peripheral systolic blood pressure
PWV: Pulse wave velocity
SV: Systolic volume
TVR: Total vascular resistance
